# Another landmark achievement by Pakistan Journal of Medical Sciences and the future challenges

**DOI:** 10.12669/pjms.38.7.6880

**Published:** 2022

**Authors:** Shaukat Ali Jawaid, Masood Jawaid

**Affiliations:** 1 Shaukat Ali Jawaid Chief Editor, Pakistan Journal of Medical Sciences Karachi - Pakistan; 2 Dr. Masood Jawaid Associate Editor, Pakistan Journal of Medical Sciences Karachi - Pakistan

As per Journal Citation Report for the year 2022 released by Clarivate on June 28^th^, Pakistan Journal of Medical Sciences has accomplished yet another landmark achievement of earning over hundred percent increase in its Impact Factor compared to the last year. Its current IF is 2.340 while last year it was 1.088.^1^ It also retains the No. 1 positon among the medical journals published from Pakistan having the highest Impact Factor. It is also heartening to note that the other two IF journals from Pakistan have also increased their Impact Factor, J Coll Physicians Surg Pak 1.024 and JPMA 1.002. As regards the number of citations which is much more important, JPMA being the oldest publication has the highest i.e 5045 as compared to Pak J Med Sci which has 4943 citations and JCPSP has 2880 citations.^1^ Our Cite Score in Scopus database has also increased and during 2021 it was 2.3 which is likely to increase further during 2022.^2^

Another six medical journals from Pakistan have also crossed the first hurdle by getting enlisted in eSCIE which is the first step towards getting IF. Calrivate has now announced that from next year all those Journals will also get an Impact Factor. It is a good news for the country. Table-I.

We are extremely grateful to our members of the Editorial Board, International Advisors and valuable reviewers for their devotion and dedication which has played a vital role in this achievement. Impact Factor, we have always maintained is just one of the criteria but not the only criteria to judge the standard of a journal. In the past there have been lot of controversy regarding the usefulness of IF but we believe that as long as there is no other authentic metric to judge it, IF will remain important and its announcement will be anxiously awaited by the editor’s community all over the world.^3^.^4^

IF it may be mentioned here is based on citation frequency of articles from a Journal. For journals in rapidly expanding field like cell biology, computers, medical education tend to get higher Impact Factor than journals in other fields like economics etc. Furthermore, some articles in a journal could be cited more frequently while others may not be cited at all, as such IF, it is said, does not accurately reflect the quality of individual articles published in a journal. Similarly, journals with more issues and articles can earn higher IF which could be misleading as it will not reflect quality of the articles. Yet another point worth noting is that Reviews usually tend to have more citations, editorials, letters, conference proceedings etc., are not counted in articles but if cited are counted as citations for the journal. As such, some editors feel that it leaves room for manipulation of ratio used to calculate the IF which could result in inflated IF in some cases.

Preview by Marie McVeigh Head of the Editorial Integrity at Web of Sciences has reported that this year’s data summarized 35,000 publications which included journals, books and conference proceedings, 2.7 million citable items and 145 million cited references indexed in Web of Sciences Core Collection.^5^ Inclusion of Early Access content in JCR metric calculation which is defined as content that is published online prior to final assignment in a completed volume and issue, besides sudden appearance of Covid19 pandemic has enabled many journals to have increased impact factor because of increased citations. The JCR 2021 contained over 25% more articles and citations as compared to the previous year.^5^Most of these items have now been incorporated into an issue in 2021 or 2022 which means they have already been counted. Hence depending on the relative volume of early access content, impact factor of some journals may decrease, hence maintaining or further increasing the IF by the journals may not be easy. It will be another challenge for us as well. The current JCR shows that over 80% of the journals showed an increase in their IF this year.

With the increased cost of production, publication charges had to be increased much against our wishes but the solution lies in having only Online Journals which offer many advantages. One hopes that the regulatory bodies in Pakistan will look at it passionately. Currently all the IF journals published from Pakistan are under tremendous pressure and the authors have to wait for too long to get their research published.^6^ If the policy of Online Journals only at least for those journals which enjoy IF and are being regularly published for the last twenty years is accepted, the journals will be able to accommodate more papers as it will not further increase their cost of production, more authors will benefit, further increase in publication charges will be checked, early publication of their papers will be possible. Not only that it will increase citations from Pakistan increasing our contribution to World medical literature tremendously.^7^ The whole world is moving towards digitalization doing away with the print version of the journals. Leading world renowned medical journals like Lancet, BMJ, JAMA all have increased their publications and they use their main journal as brand and all new publications are just online journals. Online only journal will also reduce our import of paper, plates and other items used in publishing thus saving precious foreign exchange as well. However, there remain some challenges as well because credibility has become very important in view of mushroom growth of predatory journals.^7^Policy makers and regulatory bodies like Higher Education Commission (HEC)and Pakistan Medical & Dental Council (PM&DC) need to look into these issues and try to help and facilitate the credible journals to improve their visibility and standard. One of the initiatives could be providing facility of XML file Pakistan so that more and more journals could be included in PubMed Central. This will automatically enable them to be visible on Medline as well. This require XML files for which the software is not available in Pakistan at present and journals have to get this facility from overseas. HEC in particular can look into this and they can arrange this facility.

**Table:** Pakistani Journals with Impact Factor and those included in eSCIE.

Rank	Full Journal Title	Total Cites	Journal Impact Factor 2022
1	**Pakistan Journal of Medical Sciences**	**4943**	**2.340**
2	Pakistan Veterinary Journal	1578	1.803
3	Pakistan Journal of Botany	6136	1.101
4	**JCPSP-Journal of the College of Physicians and Surgeons Pakistan**	**2880**	**1.022**
5	**Journal of the Pakistan Medical Association**	**5045**	**1.002**
6	Pakistan Journal of Pharmaceutical Sciences	3219	0.863
7	Pakistan Journal of Agricultural Sciences	1489	0.856
8	Journal of the Chemical Society of Pakistan	1110	0.698
9	Pakistan Journal of Zoology	2334	0.687
10	Khyber Medical University Journal (KMUJ)	101	N/A
11	Pakistan Journal of Analytical & Environmental Chemistry	207	N/A
12	Journal of The Pakistan Institute of Chemical Engineers	7	N/A
13	Annals of King Edward Medical University Lahore Pakistan	236	N/A
14	Pakistan Journal of Statistics and Operation Research	529	N/A
15	Pakistan Journal of Medical & Health Sciences	546	N/A
16	Pakistan Heart Journal	64	N/A
17	Rawal Medical Journal	318	N/A

***Source***: Journal Citation Report by Clarivate released on June 28th 2022.

Pakistan Association of Medical Editors (PAME) on its part has been trying to build professional capacity of editors in Pakistan. To achieve this objective, it collaborated with University of Health Sciences Lahore and started Certificate in Medical Editing (CME) and Diploma in Medial Journalism for Editors (Dip-MJE). Three CME batches have earned this certificate in which ninety-four healthcare professionals including Editors and Editorial Board members of various journals have qualified so far while the CME Batch-4 and Dip-MJE Batch-One besides second batch of Online Certificate of Medical Editing and Journalism (CMEJ) is under training. All these measures it is hoped will help improve the standard of biomedical journals published from Pakistan further in the coming years.^8^-^10^

We also plan to increase the visibility of Pakistan Journal of Medical Sciences on the social media to attract more readers and authors. Human Resource and Financial constraints have so far remained the major hurdle which we hope to overcome soon. Realizing our responsibilities of teaching and training of researchers including postgraduates, authors, reviewers, faculty members and Editorial staff of journals, we continue to organize Hands on Workshops all over the country in collaboration with various medical colleges, medical universities and hope to continue this academic activity in future as well.
